# Computationally-assisted discovery and structure elucidation of natural products

**DOI:** 10.1007/s11418-019-01321-8

**Published:** 2019-05-15

**Authors:** Alfarius Eko Nugroho, Hiroshi Morita

**Affiliations:** Faculty of Pharmaceutical Sciences, Hoshi University, Ebara 2-4-41 Shinagawa-ku, Tokyo, 142-8501 Japan

**Keywords:** Molecular networking, Density functional theory, Computer-assisted structure elucidation

## Abstract

Computer hardware development coupled with the development of quantum chemistry, new computational models and algorithms, and user-friendly interfaces have lowered the barriers to the use of computation in the discovery and structure elucidation of natural products. Consequently, the use of computational chemistry software as a tool to discover and determine the structure of natural products has become more common in recent years. In this review, we provide several examples of recent studies that used computer technology to facilitate the discovery and structure determination of various natural products.

## Introduction

Computer technology has advanced by leaps and bounds in recent years. In the hardware department, the computing power of current high-end consumer personal computers now rivals that of supercomputers available at the end of the last century. Computer hardware development coupled with the development of quantum chemistry, new computational models and algorithms, and user-friendly interfaces have lowered the barriers to the use of computation for the discovery and structure elucidation of natural products. In the present review, we provide several examples of recent studies that used spectrocopic methods in combination with computer technology to faciltate the discovery and structure determination of natural products. The review has been structured into sections based on the spectroscopic method assisted by computation.

## Tandem mass spectroscopy

Data obtained from tandem mass spectroscopy (MS/MS) studies provide information on the structure of a compound. The most basic use of computation in MS/MS data analysis is to identify a compound by matching its MS/MS data to data stored in reference spectra databases and libraries. However, as has been noted elsewhere [1–7], the reference spectra currently available are very limited, and only 1.8% of spectra in an untargeted metabolomics experiment can be annotated. Thus, other computational approaches for interpreting and predicting MS/MS data have been developed [5–12]. One method attracting the interest of those researchers working in the field of natural products is molecular networking (MN) [5] which is freely available (including a step-by-step tutorial) on the Global Natural Products Social Molecular Networking (GNPS), an open-access knowledge base for the community-wide organization and sharing of raw, processed, or identified MS/MS data [2].

**Fig. 1:**
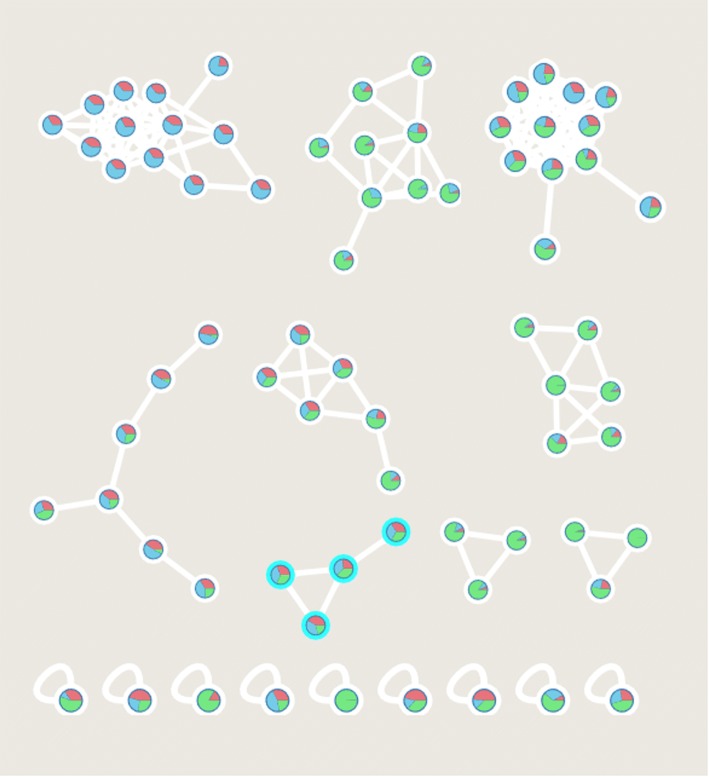
Example of molecular networking of three samples. The pie chart in a node represents the extracted ion chromatogram peak area. Identified compounds are shown as nodes with a light-blue border (color figure online)

MN is a computational method for interpreting and visualizing MS/MS data. An important aspect of MN is that it provides a visual overview of the ions of molecules in the MS/MS dataset, grouped by the similarities of their MS/MS fragmentation patterns that suggest their structural similarities, without the need of any prior knowledge regarding the chemical composition of the samples [1, 4, 5, 13]. The use of reference spectra to annotate the known compounds is also possible with MN, and these reference spectra are necessary for MN to be used as a dereplication strategy [4]. Although data visualization can be performed on GPNS, visualization of an entire molecular network is usually done with Cytoscape, an open source bioinformatics software platform [14]. In a molecular network, molecular ions are represented as nodes (usually circles), and related molecular ions are connected by edges (lines). In Cytoscape, edge thickness, node size, and node color may also be tuned to facilitate data interpretation. For example, the node color can be set to represent the data associated with the samples (e.g., the species, strains, sampling location, culture condition, or bioactivity strength), and the node size can be set to represent ion intensity or bioactivity score. Thus, the similarities and differences between two or more samples can be directly and easily observed (see Fig. 1 for an example).

**Fig. 2:**
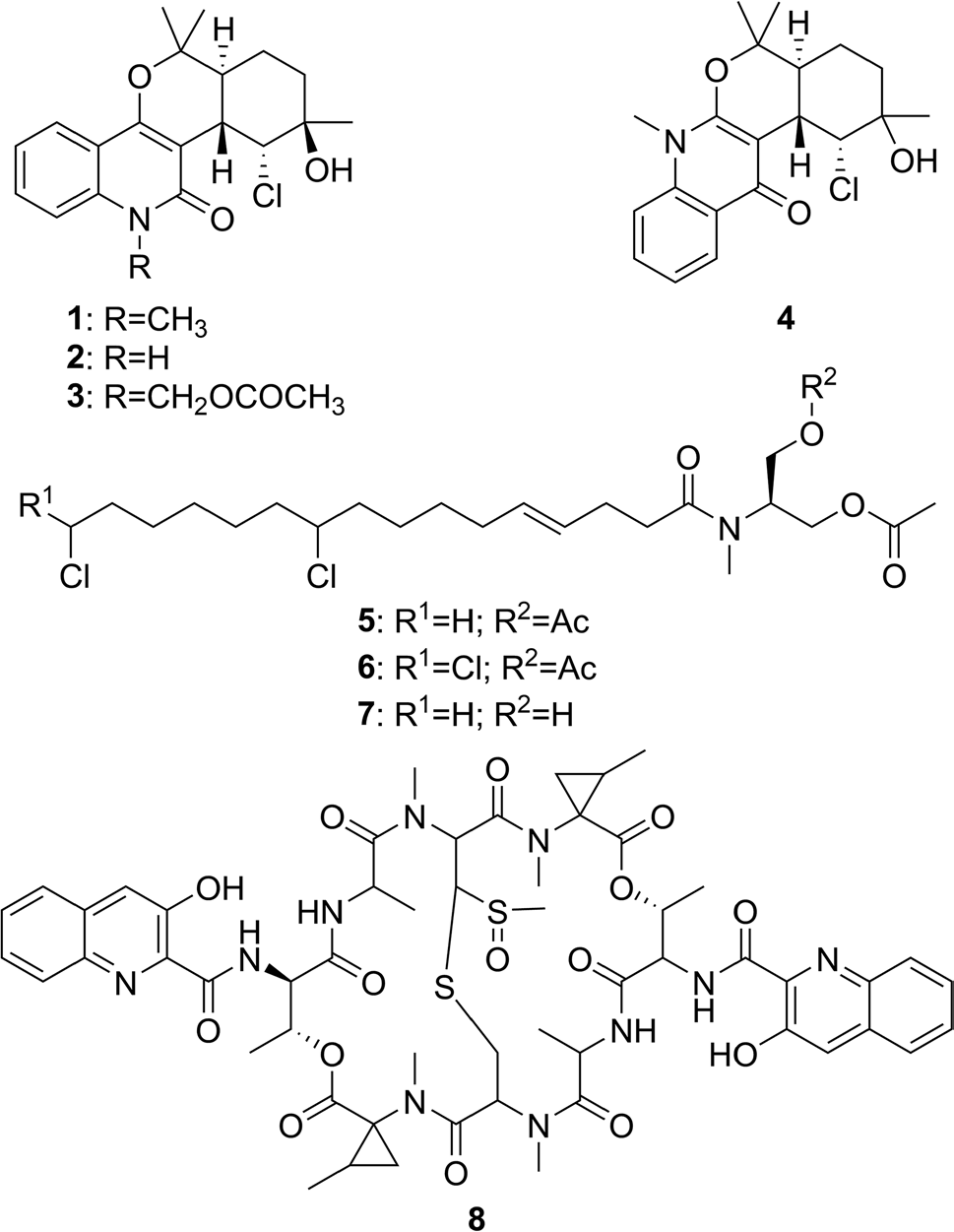
Structures of **1–8**

A growing number of reports on the successful use of MN in the discovery of natural products have been published. Application of MN to a set of 292 New Caledonian Euphorbiaceae extracts led to the identification of a group of ions specific to *Codiaeum peltatum* bark extract. Subsequent purification of the targeted compounds resulted in the isolation of four novel chlorinated monoterpenyl quinolones, namely, chloroaustralasines A–C and isochloroaustralasine A (**1–4**, Fig. 2) [15]. When bioactivity and taxonomic data were included in the analysis, application of MN to the same set of 292 New Caledonian Euphorbiaceae extracts led to the prediction of the active metabolites of *Bocquillonia nervosa* and *Neoguillauminia cleopatra* against two biological targets (Wnt signaling pathway and chikungunya virus replication), which were then verified through their isolation and bioactivity assay [16]. MN can also be used in combination with bioactivity score prediction, which is calculated by considering the relative abundance of a molecule in fractions and the bioactivity level of each fraction, as shown by the compounds with anti-chikunguya virus replication activity isolated from *Euphorbia dendroides* [17]. The combination of MN and genomic data also led to the isolation of columbamides A–C, a new class of di- and trichlorinated acyl amides with cannabinomimetic activity, from marine cyanobacteria (**5–7**, Fig. 2) [18]. Retimycin A, a new member of the quinomycin family of antibiotics, was also isolated through the use of MN and genome mining (**8**, Fig. 2) [19]. MN can also be used to explore the metabolites produced in a co-culture experiments, as shown by Dorrestein et al. [5] and Tasdemir et al. [20].

## NMR spectroscopy

### Automatic structure elucidation

Several reviews and books have been published on automatic structure elucidation, which is often referred as computer-assisted structure elucidation (CASE) [21–23]. Recently developed CASE programs, such as ACD/Structure Elucidator and Bruker CMC-se, are already able to automatize many of the steps required for the analysis and interpretation of standard one- and two-dimensional, respectively) NMR data, with minimal human interference, such as confirmation of the automatically-generated correlation table. These CASE programs also include empirical chemical-shift predictions to help in ranking the possible structures. In addition, newly designed NMR experiments have recently been performed that are evidently more machine readable, such as the “pure shift” spectra (spectra where all the ^1^H are decoupled, thus all multiplets are transformed into singlets) [24, 25]. Conventionally, CASE can only be used to predict possible planar structures. However, several attempts to include relative configuration determination inside CASE programs (CASE-3D) by including NOE [26] or residual dipolar couplings (RDC) [27, 28] data have also been reported.

CASE and CASE-3D have been successfully deployed in the structural revision or structure elucidation of natural products. Elyashberg et al. [22] reviewed CASE-based structural revision in 2010, and a more recent study involving the use of CASE-3D in the phytochemical study of *Senecio volckmannii* has been reported by Castro et al. in 2018 [29].

### Calculated anisotropic NMR data

Residual dipolar couplings, a component of anisotropic NMR data, are observed when the molecules are partially aligned in a weakly aligning medium (orienting medium that only induces a low degree of order), usually a liquid crystal or constrained polymeric gel such as PMMA [poly(methyl methacrylate)] [30] and PHEMA [poly(2-hydroxylethyl methacrylate)] [31]. Residual chemical shift anisotropy (RCSA), another component of anisotropic NMR data, is observed when the molecules are partially aligned. RDC data provide the relative orientation of different ^1^H–^13^C bonds, while RCSA data provide the relative orientations of different carbon chemical shielding tensors and, as such, are the more useful tool to study for proton-deficient molecules. The chemical shift tensor information necessary for RCSA analysis can be obtained using density functional theory (DFT) calculations. Several reviews on the use of RDC and RCSA for the determination of relative configuration of small organic molecules have been published [27, 32–37]. More importantly, a protocol on the use of anisotropic NMR parameters for the structure elucidation of small organic compounds has recently become available [38].

### NMR parameter prediction by DFT calculation

**Fig. 3:**
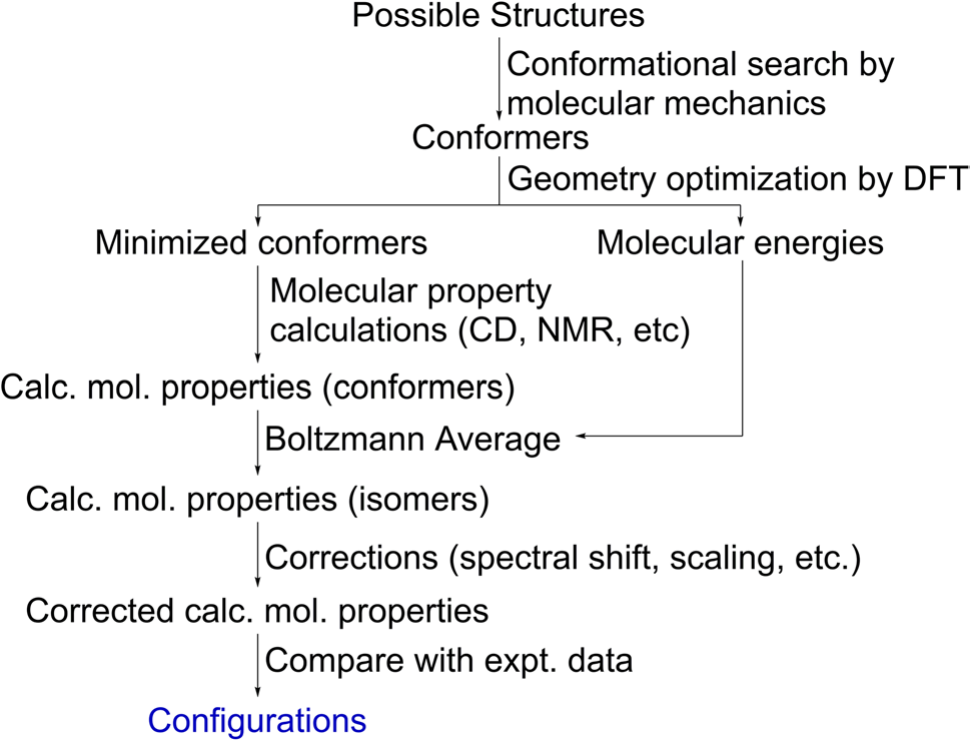
Steps in molecular property calculations by density functional theory (*DFT*)

In general, property prediction using DFT calculations involves (1) a conformational search by Monte Carlo methods using molecular mechanics [Merck molecular force field (MMFF) 94, among others] and/or semi-empirical methods [Austin Model 1 (AM1), among others]; (2) geometry optimization at the DFT level; (3) molecular property calculations at the DFT level; (4) Boltzmann-weighting of the molecular properties; (5) correction of the molecular properties (by wavelength shifting, chemical shift scaling, etc.); and (6) comparison of the experimental and corrected calculated properties (Fig. 3). It should be noted that two DFT calculations [steps (2) and (3)] can be performed on different levels, i.e., different density functional approximations (DFAs) and/or basis-sets with/without solvent effect modeling. The calculation level is often expressed as [Functional 1]/[Basis set 1]//[Functional 2]/[Basis set 2], which means that the property calculation was performed by using Functional 1 and Basis set 1 on a geometry optimized by using Functional 2 and Basis set 2. In addition, several methods for calculating the DFT-NMR are currently available, such as the gauge-including atomic orbitals (GIAO) method, the individual gauge for localized orbitals (IGLO) method, and the continuous set of gauge transformation (CSGT) method. Among these three methods, GIAO is the most commonly used method.

DFT calculations can be performed by using programs such as the Gaussian, Turbomole, NWChem, ORCA, or Spartan program. The accuracy of the DFT calculations mainly depends on the basis set and DFAs used for the calculations. Over the past decades, as a consequence of the unknown true density functional, many DFAs have been developed to provide better accuracy and/or lower computational costs (shorter computational time). Some of the DFAs were developed with a specific use in mind, such as for kinetics, thermodynamics, or transition metals, and may not perform well in calculations of properties other than the intended one.

Goerigk et al. [39] recently assessed the performance of 217 variations of London dispersion-corrected and -uncorrected DFAs of the barrier heights, basic properties, and reaction energies for small systems, the reaction energies for large systems and the isomerization reaction, intramolecular non-covalent interactions, and intermolecular non-covalent interactions, and reported a recommended DFA for each specific purpose and for the general purpose. The use of dispersion correction is generally recommended in order to obtain better relative conformational energies and geometries of organic compounds [39]. The best general-purpose hybrid DFAs are ωB97X-V, M052X-D3(0), ωB97X-D3, and M06-2X-D3(0). Regarding general-purpose conventional hybrid DFAs, the recommended DFA is PW6B95-D3(BJ) (dispersion correction with BJ-damping of PW6B95 DFA). The more popular hybrid functional B3LYP is only ranked 18th. Among the low-cost generalized gradient approximation DFAs, the best three DFAs are revPBE-D3(BJ), B97-D3(BJ) and OLYP-D3(BJ). D3(0) and D3(BJ) are dispersion correction methods developed by Grimme et al. [40] with zero damping or Becke and Johnson (BJ) damping [41], respectively.

For DFT calculation in general, the use of a bigger basis set and inclusion of solvent effect modeling, such as IEF-PCM [42–44] and COSMO [45], will lead to better accuracy. However, it is important to note that while the inclusion of solvent effect modeling will not significantly increase the calculation time, the use of a larger basis set will significantly increase it.

Magnetic shielding tensors are obtained by using DFT calculations on NMR data. The anisotropic tensors are used in RCSA calculations, and the isotropic shielding constants are used in chemical shift calculations. Generally, the isotropic shielding constants are converted and corrected to chemical shifts by using either linear scaling (Eq. 1) or reference compounds (Eq. 2):

1 $$\delta_{i} = \frac{{{\text{intercept}} - \sigma_{i} }}{\text{slope}},$$

2 $$\delta_{i} = \sigma_{\text{ref}} - \sigma_{i} + \delta_{\text{ref}} ,$$

where *δ*_*i*_ is the calculated chemical shift of the nucleus of interest, *δ*_ref_ is the experimental chemical shift of the reference nucleus, and *σ*_*i*_ and *σ*_ref_ are the calculated isotropic shielding constant of the nucleus of interest and the reference nucleus, respectively. It should be noted that both *σ*_*i*_ and *σ*_ref_ must be computed at the same level of theory. In addition, the scaling factors (intercept and slope) are empirical values obtained from the linear regression of the calculated isotropic shielding tensors from a set of reference compounds, at a given calculation level, which are plotted against their experimental chemical shifts. Therefore, each calculation level will have its own distinct scaling factors [46, 47]. A database of scaling factors collected by Tantillo and coworkers is available on the CHESHIRE CCAT website [48].

As stated above, each calculation level will have its own distinct scaling factors. Consequently, new scaling factors need to be generated for each new calculation level before the general researchers studying the natural product can use it. A more general approach, Eq. 2, is the use of reference compounds. It has been shown that the use of any single reference compound, e.g., tetramethylsilane (TMS), leads to unsatisfactory results [49, 50], leading to the proposal that multiple reference compounds [multi-standard methodology (MSTD)] be used to improve calculation accuracy [49–51]. The use of methanol for sp^3^ environments and benzene for sp^2^ environments [49, 51] or the use of multiple reference compounds possessing an environment similar to that of the compound of interest [50] has been proposed. A combination of both scaling factors and multiple reference compounds has also been proposed [52]. Finally, it is important to note that when using new calculation levels, which may provide better relative conformational energies, better geometries, or faster calculation times, the MSTD approach is more straightforward to implement than the scaling factors approach.

To evaluate the goodness-of-fit between the experimental and calculated chemical shifts, mean average error (MAE), or corrected mean average error (CMAE) for scaled data, is often used. The CP3 and DP4/DP4+ parameters are alternatives to evaluating the goodness-of-fit [53–55]. The CP3 parameter is designed to assign two sets of experimental data to two sets of structures, and the DP4/DP4+ probability parameter is designed to determine the most plausible isomer among many. Another alternative is the use of an artificial neural network for pattern recognition developed by Sarotti [56].

For most researchers on natural products, the big question is “what is the recommended calculation level?”

For the scaled chemical shift calculation, considering the limited number of available scaling factors, the use of B3LYP/6–31+G(d,p) or M06-2X/6–31+G(d,p) for the geometry optimization step combined with GIAO-mPW1PW91/6–311+G(2d,p) or GIAO-PBE0/6–311+G(2d,p) for the NMR calculation is recommended by Tantillo and coworkers for high-accuracy calculations [48]. On the other hand, the recommended level for the low-cost calculation is GIAO-B3LYP/6–31+G(d,p)//B3LYP/6–31G(d) [48]. It should be noted that for all recommended calculation levels, the implicit solvent model for chloroform using the conductor-like polarizable continuum model (CPCM) or solvation model density (SMD) is included at the NMR calculation step.

For the MSTD approach, Sarotti and Pellegrinet noted that the quality of the calculated chemical shifts is much less dependent on the calculation level [49, 51]. The use of solvent effect modeling increased the accuracy of the calculations. Moreover, the use of triple-ζ-basis sets, such as cc-pVTZ or 6–311+G(d,p), generally gave slightly better results than did the double-ζ-basis sets, such as 6–31+G(d). However, the computationally faster double-ζ-basis sets still gave good results. For the DFA part, between mPW1PW91, B3LYP, and WP04, Sarotti suggested the use of mPW1PW91 [49, 51].

**Fig. 4:**
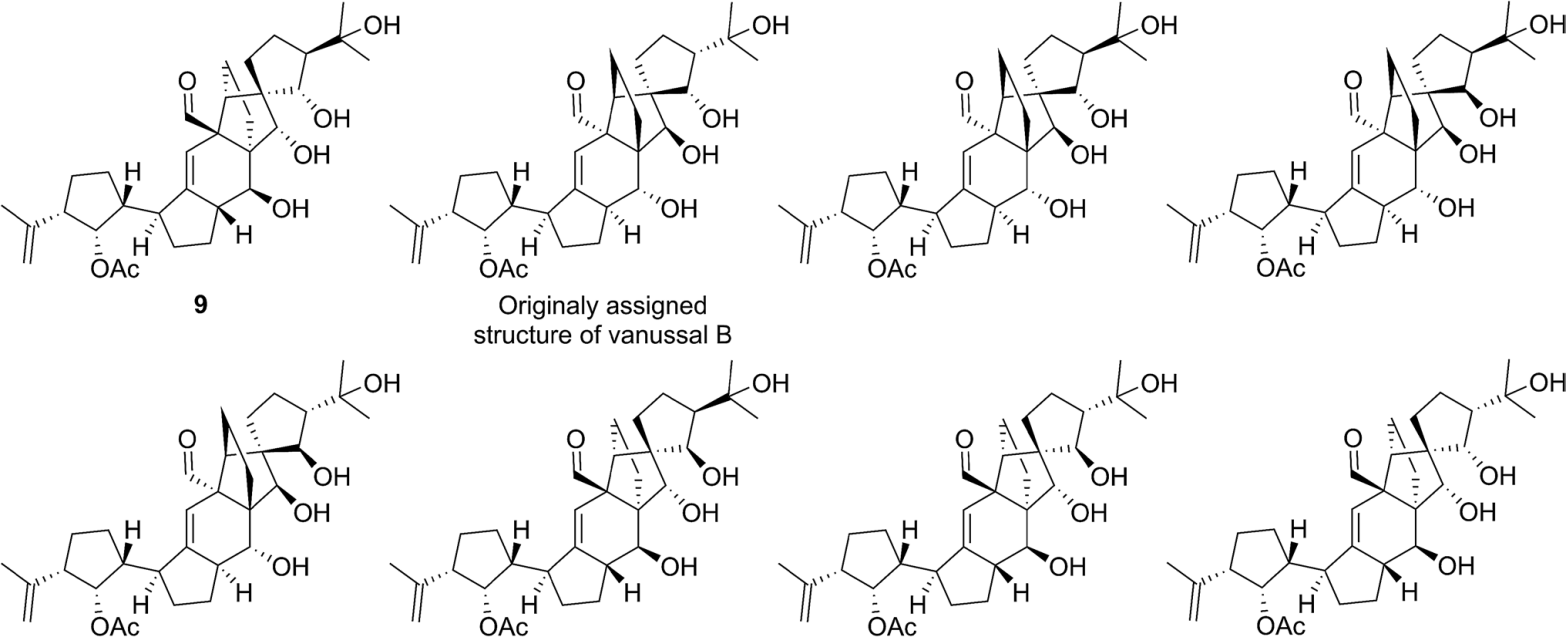
Structures of vannusal B (**9**) and the diastereomers synthesized for its structural revision

**Fig. 5:**
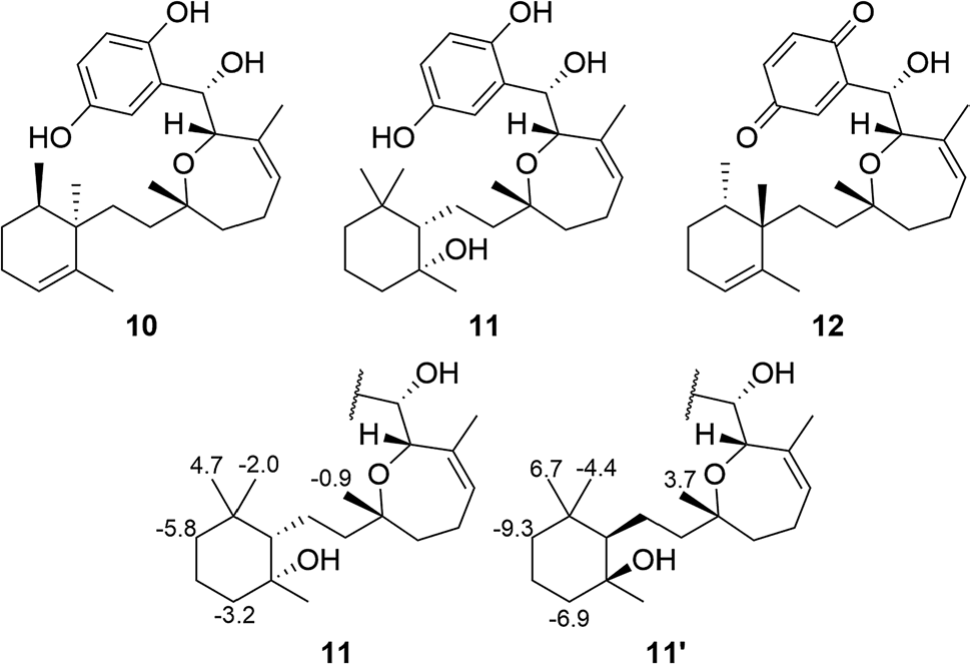
Structures of **10–12**, and comparison of Δ*δ*_C_ (*δ*_Ccalc_–* δ*_Cexpt_) between **11** and its diastereomer.

There are many reports on the use of DFT-calculated NMR chemical shifts for the structure revision or structure elucidation of natural products. Willoughby and coworkers recently published the Phyton scripts, which are helpful in automating many aspects of the DFT calculations of NMR chemical shifts [57]. Bagno and coworkers showed that the structural revision of vanussal B (**9**, Fig. 4) could have been greatly simplified by the use of chemical shifts calculated using DFT [58]. DFT-calculated NMR has been used to determine the relative configuration of various natural products, with recent reports on halioxepines A–C isolated from marine sponges of the genus *Haliclona* (**10–12**, Fig. 5) [59], xylomolones A–B isolated from *Xylocarpus moluccensis Haliclona* [60], bisleuconothine B isolated from *Leuconotis griffithii Haliclona* [61], ceramicine N isolated from *Chisocheton ceramicus* [62], and walsogyne B isolated from *Walsura chrysogyne* [63]. It interesting that even in difficult cases such as **10–12**, where two stereoclusters are separated by two methylenes, DFT NMR calculations can accurately predict the relative configurations of the product.

## Electronic circular dichroism, vibronic circular dichroism, optical rotation (or) and optical rotatory dispersion

An often challenging problem in the structure elucidation process of natural products is the determination of the absolute configuration (AC) of those products. Various approaches have been adopted to solve this problem, including X-ray crystallography, chiroptical methods, and NMR anisotropy methods, but each approach has its own specific limitations. Recently, comparison of the time-dependent density functional theory (TDDFT)-calculated chiroptical data with experimental chiroptical data has become commonly accepted approach to determine the AC of natural products. The principle of the determination of the ACs of natural products by TDDFT calculation is relatively simple: basically, the calculated chiroptical data are compared with the experimental data, and if the two sets of data are very similar to each other, then highly reliable assignment can be obtained. The use of TDDFT-calculated chiroptical data for the determination of ACs has been extensively reviewed and discussed in tutorials [64–69].

Regarding the calculation level for the geometry optimization calculations, a combination of B3LYP/6–31G(d) or BP86/def–SVP level of theory is often used. However, as mentioned above, there are other DFAs that should perform better. And although double-ζ-basis sets, such as def2-SVP or 6–31G(d), may give sufficient accuracy, triple-ζ-basis sets, such as def2-TZVP or 6–311+G(d,p), should give better results. The use of dispersion correction (D3) and solvent effect modeling are also recommended. As a final note, vibronic circular dichroism (VCD) calculations are more prone to inaccuracies arising from the geometry optimization calculations.

Regarding the calculation levels for the chiroptical data calculations, the use of basis sets with polarization and diffuse functions, such as 6-31G* or aug-cc-pVDZ, is very important in the calculation of electronic circular dichroism (ECD). The choice of DFA is less clear for ECD calculations, but the recommendation of Goerigk and Grimme [39], mentioned earlier in this review, may provide a clue. Indeed, although the popular DFAs, such as B3LYP and BP86, may be sufficient in many cases, the DFA ωB97X-D3 often give better results [65, 66]. For VCD calculations, the low-cost B3LYP/def2-SVP and B3LYP/6–31G(d) DFAs, or the more costly B3LYP/def2-TZVP are usually sufficient [65, 68]. For optical rotation and/or optical rotatory dispersion calculations, combinations of the B3LYP or PBE0 hybrid functional with aug-cc-pVDZ are usually sufficient. Since chiroptical data are solvent dependent, the use of solvent effect modeling is highly recommended.

The results of TDDFT calculations of UV/ECD are excitation energies and their corresponding oscillator strength and rotatory strength. The oscillator strengths are used to simulate the UV curve, and rotatory strengths are used to simulate the ECD curve. Both oscillator strength and rotatory strength can be calculated by using either the dipole–velocity gauge or the dipole–length gauge, but the use of the dipole–length gauge generally gives better results [70, 71]. The calculated oscillator strength and rotatory strength values are generally converted to the UV/ECD curve by using a Gaussian distribution function (see reference [64] for more details). The calculated UV spectra are then shifted to conform to the experimental UV spectrum, and the same shifts are also applied to the corresponding calculated ECD spectra before the calculated ECD spectra are compared with the experimental ECD of the natural product in question.

In contrast to UV/ECD calculations, the results of IR/VCD calculations are generally converted to the IR/VCD curve by using a Lorentzian distribution function, and instead of shifting, calculated IR/VCD spectra are usually scaled by a factor in the range of 0.97–0.98 [68].

There are at least several hundreds of publications reporting the use of calculated chiroptical data to assign AC to a broad range of compounds (see references [64–69] for some examples; reference [64] is available as an open access article in this journal). It can be argued that the use of calculated chiroptical data has become a routine part of procedure used in the AC assignments of natural products. In our search for bioactive compounds, we have also used calculated chiroptical data to assign the AC of isolated compounds [61, 63, 64, 72–81].

## Conclusion

Computational methods can be used in practically all of the steps of the discovery and structure determination of natural products, and they have been shown to be a very useful tool in such studies. Many studies have used such methods; for example, Hamann and co-workers used MN and DFT-calculated NMR chemical shifts and ECD spectra in the discovery and assignment of aleutianamine, a new class of pyrroloiminoquinone alkaloids isolated from *Latrunculia austini* [82]. There have been recent effort and initiatives to make raw MS/MS and NMR data publicly available [2, 83]. The public availability of these data is very important in the development of new computational methods, in particular those that involves machine learning, such as CSI:FingerID [6] and the artificial neural network pattern recognition developed by Sarotti [56]. It can be expected that in the future most discoveries and structure elucidations of natural products will be fully assisted by computational methods.
