# Correction to: Computationally-assisted discovery and structure elucidation of natural products

**DOI:** 10.1007/s11418-019-01340-5

**Published:** 2019-06-28

**Authors:** Alfarius Eko Nugroho, Hiroshi Morita

**Affiliations:** Faculty of Pharmaceutical Sciences, Hoshi University, Ebara 2-4-41 Shinagawa-ku, Tokyo, 142-8501 Japan

## Correction to: Journal of Natural Medicines 10.1007/s11418-019-01321-8

The article Computationally-assisted discovery and structure elucidation of natural products, written by Alfarius Eko Nugroho and Hiroshi Morita, was originally published electronically on the publisher’s internet portal (currently SpringerLink) on 15 May 2019 without open access.

With the author(s)’ decision to opt for Open Choice the copyright of the article changed on 25 June 2019 to © The Author(s) 2019 and the article is forthwith distributed under the terms of the Creative Commons Attribution 4.0 International License (http://creativecommons.org/licenses/by/4.0/), which permits use, duplication, adaptation, distribution and reproduction in any medium or format, as long as you give appropriate credit to the original author(s) and the source, provide a link to the Creative Commons license and indicate if changes were made.

The original article has been updated.

